# Cell-autonomous and non-autonomous roles of *daf-16* in muscle function and mitochondrial capacity in aging *C. elegans*

**DOI:** 10.18632/aging.101914

**Published:** 2019-04-24

**Authors:** Hongning Wang, Phillip Webster, Lizhen Chen, Alfred L. Fisher

**Affiliations:** 1 Division of Geriatrics, Gerontology, and Palliative Medicine, Department of Medicine, UTHSCSA, San Antonio, TX 78229, USA; 2 Center for Healthy Aging, UTHSCSA, San Antonio, TX 78229, USA; 3 Department of Cell Systems and Anatomy, UTHSCSA, San Antonio, TX 78229, USA; 4 GRECC, South Texas VA Healthcare System, San Antonio, TX 78229, USA; 5 Division of Geriatrics, Gerontology, and Palliative Medicine, Department of Medicine, University of Nebraska Medical Center, Omaha, NE 68198, USA; * Equal contribution

**Keywords:** muscle, aging, mitochondria, *daf-2*, *daf-16*

## Abstract

Sarcopenia, defined as the loss of skeletal muscle mass and strength, contributes to disability and health-related conditions with aging. In vitro studies indicate that age-related mitochondrial dysfunction could play a central role in the development and progression of sarcopenia, but because of limitations in the methods employed, how aging affects muscle mitochondrial function in vivo is not fully understood. We use muscle-targeted fluorescent proteins and the ratiometric ATP reporter, ATeam, to examine changes in muscle mitochondrial mass and morphology, and intracellular ATP levels in *C. elegans*. We find that the preserved muscle function in aging *daf-2* mutants is associated with higher muscle mitochondrial mass, preserved mitochondrial morphology, and higher levels of intracellular ATP. These phenotypes require the *daf-16*/FOXO transcription factor. Via the tissue-specific rescue of *daf-16*, we find that *daf-16* activity in either muscle or neurons is sufficient to enhance muscle mitochondrial mass, whereas *daf-16* activity in the muscle is required for the enhanced muscle function and mobility of the *daf-2* mutants. Finally, we show through the use of drugs known to enhance mitochondrial activity that augmenting mitochondrial function leads to improved mobility during aging. These results suggest an important role for mitochondrial function in muscle aging.

## Introduction

During aging all people lose muscle mass and strength with the peak strength and muscle mass being observed between the late 20’s and early 40’s (reviewed in [1]). The term sarcopenia is often used to refer to these losses, and the study of sarcopenia is of interest both at a scientific and clinical level. In particular, the loss of muscle mass and muscle strength is connected with the development of geriatric phenomena including reduced gait speed, mobility limitations, need for assistance with daily activities, and falls. As a result of the intertwined losses of muscle mass and strength, and the associated functional declines, definitions of the condition have been proposed that focus on from one to all of these aspects [1]. While the exact percentages vary depending on the definition used, the population sampled, and the technique used for assessment, clinically meaningful levels of sarcopenia are thought to affect roughly 30% of individuals over 60 years of age and up to more than 50% of people over 80 years [2]. Unfortunately, the adverse effects of muscle aging produce both human and financial costs. For example, one study estimated that if 45% of the older U.S. population is affected by sarcopenia, then the estimated direct healthcare costs attributable to sarcopenia in the United States was $18.5 billion in 2000, which is approximately 1.5% of the total healthcare expenditures for that year [3]. Conversely, if the prevalence of sarcopenia could be reduced by just 10% then this could lead to healthcare cost savings of $1.1 billion (in 2000) per year in the U.S. alone [3]. Given growing numbers of older people in both the U.S. and worldwide, both the numbers of individuals who develop sarcopenia and the impact on health and wellness are likely to increase in the coming years.

Despite the fairly high prevalence of sarcopenia, our knowledge of the mechanisms involved is incomplete, and consequently there are few proven treatment approaches for the condition other than regular weight-bearing exercise. Both clinical and basic research has provided evidence for multiple potential mechanisms ranging from losses of motor neurons, hormonal changes, and alterations in the function of muscle stem cells, called satellite cells, that alone or together could cause or worsen the age-related changes in the muscle (for recent review see [4]). Among these mechanisms, multiple studies have suggested that mitochondrial dysfunction could either be a hallmark of muscle aging or even play a causal role in the process (for review see [5,6]). The adverse effects of aging on muscle mitochondria include changes such as a reduction in mitochondrial DNA and increase in mitochondrial DNA damage [7–9]; a reduction in the coupling of mitochondrial electron transport activity to ATP production [10,11]; declines in mitochondrial quality control due to decreased removal of mitochondria by mitophagy [10]; changes in the number, size, and orientation of the mitochondria in the myocytes [12]; and an increased tendency of older mitochondria to promote myocyte apoptosis [10]. However, the relative importance of each of these adverse changes in the development and progression of sarcopenia is unclear. Another limitation of these results is that the data are generated from samples removed from either animals or human subjects and either the removal or subsequent processing might alter the structure or function of the mitochondria compared to their in vivo properties. For example, the subcellular fractionation of muscle tissue to isolate mitochondria has been reported to selectively damage the mitochondria from older individuals [13].

The non-parasitic nematode *Caenorhabditis elegans* has been shown to also develop declines in muscle mass and function during aging [14,15]. The cause of these declines is unclear, but adverse effects of the aging process on both the muscle and motor neurons may play a role [14,16]. The use of *C. elegans* as a model of muscle aging is attractive due to the short lifespan of the animals, the amenability to genetic manipulation and RNAi treatment, and the optical transparency of the animals which allows the muscles to be observed in vivo via non-invasive imaging approaches. We and others have shown that mutations affecting the *daf-2*/IGFR insulin/insulin-like growth factor receptor both extend lifespan and delay the onset of age-related declines in muscle function in these worms [14,15,17]. Hence, the study of muscle aging in *C. elegans* permits the examination of aging effects on components of the muscle, such as the mitochondria, and also permits the parallel examination of each component in animals with altered rates of muscle aging.

In this work, we examine the effects of aging on in vivo muscle mitochondrial mass and function. We adapted the ATeam ratiometric reporter for use in *C. elegans* to examine mitochondrial function by assessing the relative ATP levels in myocytes in vivo [18]. We find that mitochondria dysfunction might play a causal role in muscle aging as we find that the treatment of worms with both riboflavin [19] and methylene blue [20], which can enhance the activity of dysfunctional mitochondria, increase muscle function. We then use the *daf-2*/IGFR mutant, which also exhibits preserved mobility [14,15,17] and enhanced mitochondrial function during aging [21], to examine the interplay between the muscle and nervous system with regards to mitochondrial activity and muscle function during aging. Via the use of tissue-specific *daf-16* transgenes, we unexpectedly find that restoring *daf-16*/FOXO in either body wall muscle or neurons is able to attenuate the loss of muscle mitochondria whereas *daf-16* only reduces age-related declines in muscle mitochondrial activity and muscle function when restored in the muscle. These findings suggest that both cell autonomous and non-cell autonomous mechanisms could contribute to the beneficial effects of *daf-2*/IGFR mutations on muscle mitochondria and muscle function during aging.

## RESULTS

### Mitochondria enhancing drugs promote mobility during aging

**Figure 1:**
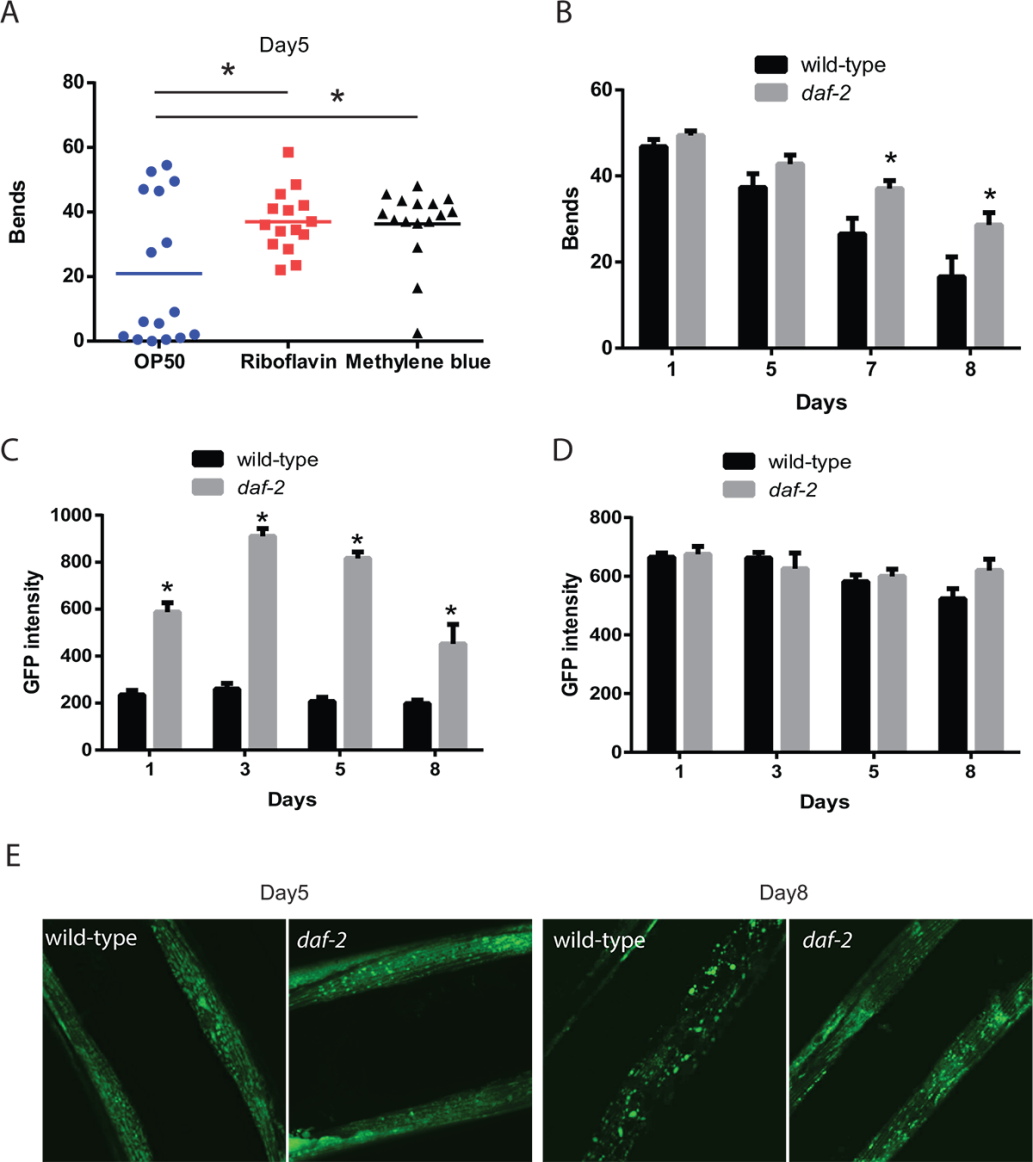
**Increased mitochondrial capabilities are associated with enhanced mobility during aging.** (**A**) Animals treated with riboflavin and methylene blue which can increase the activity of dysfunctional mitochondria show increased mobility compared to control treated animals. The mobility of wild-type adult day 5 animals, treated with or without mitochondrial activators riboflavin (2.6 mM) and methylene blue (75 mM), was assessed by the measurement of thrashing behavior in liquid. The scatter plot graphs show the average numbers of body bends during a 30 second period. N = 12 for all genotypes and ages. * represents p < 0.05 by *t*‐test. (**B**) Reductions in *daf-2* insulin-like signaling in the *daf-2/*IGFR mutant result in preserved mobility during aging. The mobility of aging wild-type and *daf-2(e1371)* mutant animals was assessed through the measurement of thrashing behavior in liquid. The bar graphs show the average numbers of body bends during a 30 second period on days 1, 3, 5, and 8 of adulthood. N = 12 for all genotypes and ages. * represents p < 0.05 by *t*‐test. (**C**) *daf-2* mutants exhibit increased muscle mitochondrial mass as shown by digital imaging and quantitation of a muscle-expressed mitochondrial targeted GFP protein. Bars represents average muscle GFP fluorescence on adult days 1, 3, 5, and 8 in *daf-2* mutant and wild-type animals. N >12 animals for all genotypes and ages. * represents p < 0.05 by *t*‐test. (**D**) *daf-2* mutant and wild-type animals show similar expression of muscle filaments as revealed by digital imaging and quantitation of a muscle-expressed MYO-3::GFP fusion protein. N >12 animals for all ages and genotypes. (**E**) Confocal images showing muscle mitochondria in animals expressing a mitochondrial-localized GFP reveal that on adult day 5, the mitochondrial in *daf-2* mutant animals show a well-organized interconnected reticulum of mitochondria. In wild-type animals, the mitochondria network shows some gaps and consists of fewer mitochondria as a longer exposure time is needed to capture an image with similar brightness to the *daf-2* mutant. By adult day 8, the wild-type animals show a significant breakdown of the network with the formation of large abnormally-shaped mitochondria. In contrast, the filamentous network is still largely intact in the *daf-2* mutants.

Mitochondria are the primary producers of intracellular energy, which can be used for a number of processes in myocytes, including muscle contraction, maintaining membrane polarization, protein synthesis, or maintaining proteostasis, and hence dysfunctional mitochondria could play a key role in age-related declines in muscle function. During aging, *C. elegans* exhibits declines in mobility with regards to both crawling on solid media and thrashing-like swimming movement in liquid [14,17,22–24]. To evaluate whether enhanced mitochondrial activity can improve muscle function, we tested the effects of the mitochondrial activators, riboflavin and methylene blue on the age-related declines in muscle function. Both of these drugs have been shown to enhance the respiratory activity of dysfunctional mitochondria due to aging (methylene blue [20]), or due to genetic mutations affecting the electron transport chain (riboflavin [19]). When we treated wild-type animals with these drugs starting on day 1 of adult life, we found that the mobility of treated day 5 animals, as measured by the number of body bends made by the animals during 30 seconds of observation, was increased compared to untreated control animals (Figure 1A). This finding suggests that enhancing mitochondrial function can enhance muscle function in older animals though this could be mediated by effects either in the muscle or another tissue.

### The *daf-2* pathway enhances muscle function and muscle mitochondrial mass

Mutations affecting the *daf-2*/IGFR gene both increase lifespan and delay the declines in muscle function and mobility [14,15,17,22]. The *daf-2* mutant animals also exhibit improved mitochondrial function during aging [21]. Hence, we compared the effects of the *daf-2* mutation on both mobility and aspects of mitochondrial structure and function to determine if these may be linked. We found that the swimming behavior of worms declined with aging in both wild-type and *daf-2* mutants, but the rate of decline appears to be lower in the *daf-2* mutants (Figure 1B). In particular, the difference in the number of body bends while swimming between the wild-type animals and *daf-2* mutants becomes initially apparent on adult day 5 and then grows on day 7 and day 8 of adulthood.

Using the same experimental conditions, we also examined how muscle mitochondrial mass, as defined by the average fluorescence produced by a mitochondrial-localized GFP, changed during aging and was affected by the *daf-2* mutation. In wild-type animals, there was a slight decline in muscle mitochondrial mass during aging (Figure 1C), whereas there was a marked increase in mitochondrial mass in young adult *daf-2* worms, which increased further in adult day 3 and day 5 worms before showing a decline in day 8 adults. Importantly, the mitochondrial mass of the *daf-2* mutants was always greater than that observed in the wild-type animals (Figure 1C). These changes were consistent with the increases in mitochondrial length and area seen in *age-1*/PI3K mutants compared to wild-type worms during aging [25]. To test whether the change in the mitochondrial-localized GFP reflected either a global increase in protein stability or GFP fluorophore activity in the *daf-2* mutants, we examined the expression of a MYO-3::GFP fusion protein which localizes to the contractile filaments of the myocytes. In contrast to the increase in the mitochondrial-localized GFP seen in the *daf-2* mutants, we found little difference in the fluorescence from the MYO-3::GFP protein between genotypes (Figure 1D). These findings suggest that there is an increase in mitochondrial mass in young adult *daf-2* mutant worms, and that these increases occur during the period of preserved muscle function and mobility.

In addition to the overall increase in muscle mass seen in the *daf-2* mutants, there were also changes in mitochondrial morphology when viewed with confocal microscopy. In day 5 adults, the mitochondria in both wild-type and *daf-2* mutant worms are aligned with the myofilaments and show a tubular structure (Figure 1E). However, the wild-type muscle mitochondria do show some evidence of early mitochondrial fragmentation. Importantly in the wild-type animals, we needed to increase the camera exposure time used to be able to capture images with similar levels of GFP fluorescence to those seen in the *daf-2* mutants. By adult day 8, the wild-type animals show severe mitochondrial fragmentation (Figure 1E) while the *daf-2* mutants still maintain the earlier tubular structure. Our findings are consistent with prior work showing a similar deterioration in muscle mitochondrial structure in wild-type animals and preservation of this structure in *age-*1/PI3K mutants [25]. Together our results show both declines in the mass and overall structure of the mitochondria in aging wild-type worms compared to *daf-2* mutants. Importantly, the timing of these changes in the *daf-2* mutants during aging corresponds to a period of preserved mobility compared to wild-type N2 animals.

### The enhanced mitochondrial mass leads to an increase in muscle ATP levels

**Figure 2:**
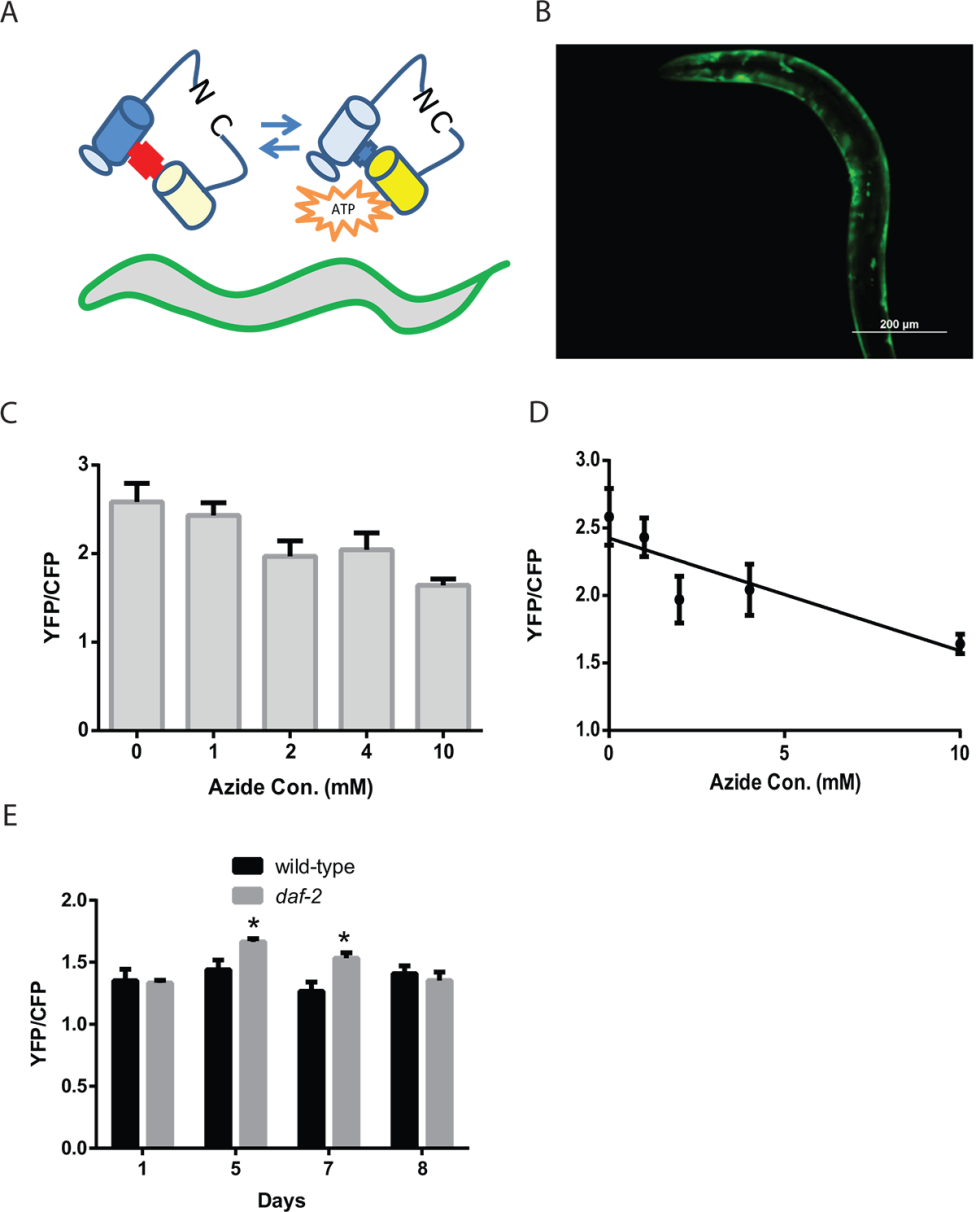
**Reduced *daf-2* insulin-like signaling increases ATP levels *in C. elegans* muscle.** (**A**) Cartoon of the ATeam ratiometric reporter (*A*denosine 5’-*T*riphosphate indicator based on *E*psilon subunit for *A*nalytical *M*easurement) which consists of the cyan fluorescent protein derivative mseCFP and the yellow fluorescent protein mVenus flanking the ε-subunit of from the F_0_F_1_-ATP synthase from *Bacillus subtilis*. The subunit from the F_0_F_1_-ATP synthase binds ATP and thereby changes in the spatial arrangement of the fluorophores which then results in alterations in the excitation of the yellow fluorophore by the cyan fluorophore *via* FRET. (**B**) Fluorescence image of a wild-type worm expressing the ATeam reporter under the control of the *myo-3* promoter in the body-wall muscle. Image was captured using a GFP filter set which can visualize mVenus fluorescence. Bar: 200 µm. (**C**) ATeam shows a reduction in the ratio of mVenus to mseCFP fluorescence (YFP/CFP) when ATP levels are reduced by treatment with the mitochondrial inhibitor sodium azide. Worms expressing ATeam in the muscle were treated with increasing doses of sodium azide for 1 hour before being mounted and digitally imaged. N >12 for all treatments. These data also exhibit a linear decline when plotted as an X-Y graph (**D**). (**E**) The *daf-2* mutants have increased muscle ATP levels on adult day 5 and day 7 as shown by the imaging of wild-type and *daf-2* mutant animals expressing the ATeam reporter. Each bar represents the average ATeam YFP/CFP ratio from the muscles of *daf-2* mutant and wild-type animals on the indicated days of adulthood. N >12 for all ages and genotypes. * represents *p* < 0.05 by *t*‐test.

An important experimental advantage of *C. elegans* is the optical transparency of the animals which allows aspects of cell behavior to be observed in vivo via the use of fluorescent proteins [26]. To examine whether the enhanced mitochondrial mass and structure seen in the *daf-2* mutants resulted in changes in muscle cell physiology, we developed transgenic worms expressing the ratiometric reporter ATeam, which shows reproducible changes in FRET-derived fluorescence in response to local ATP concentrations [18]. ATeam, or *A*denosine 5’-*T*riphosphate indicator based on *E*psilon subunit for *A*nalytical *M*easurement, consists of the cyan fluorescent protein derivative mseCFP and the yellow fluorescent protein mVenus flanking the ε-subunit of the F_0_F_1_-ATP synthase from *Bacillus subtilis* (Figure 2A). The binding of ATP to the ε-subunit brings the mseCFP and mVenus fluorophores closer together and enhances the efficiency of energy transfer from mseCFP to mVenus via FRET. Hence the amount of fluorescence emitted by ATeam at the emission wavelength for mseCFP (475 nm) versus the emission wavelength for mVenus (527 nm) when mseCFP is excited with 435 nm wavelength light reflects the amount of ATP bound to the reporter protein (Figure 2A). We targeted this transgene to muscle via the use of the *myo-3* promoter (Figure 2B).

To test whether the muscle-expressed ATeam reporter responds to changes in intracellular ATP levels, we treated worms carrying the reporter with increasing concentrations (0, 4, 7, and 10 mM) of the mitochondrial complex IV inhibitor sodium azide. Prior work has shown that azide treatment lowers ATP levels in worms, and that a 10 mM dose will produce a roughly 50% decline in whole-animal ATP levels [27]. Following a 1 hour treatment with each azide dose, we saw a progressive decline in the FRET-coupled mVenus fluorescence relative to the uncoupled CFP fluorescence, as denoted by the ratio YFP/CFP (Figure 2C and 2D). Specifically, the 10 mM azide dose produced a roughly 40% decline in this ratio which indicates that the ATeam reporter responds to real-time changes in cellular ATP levels.

We then used the ATeam reporter to follow muscle ATP levels in aging wild-type and *daf-2* mutant animals. The ATeam reporter showed a similar ratio of mVenus/mseCFP fluorescence in young adult animals, whereas the adult day 5 and day 7 *daf-2* mutant worms showed an increase in the YFP/CFP ratio relative to wild-type animals, which is suggestive of an increase in cellular ATP levels (Figure 2E). Interestingly, these increases are only seen on day 5 and day 7 even though both the day 1 and day 8 animals also exhibit increased mitochondrial mass compared to the wild-type animals (Figure 1C). In addition, we also observed that the wild-type animals show little change in muscle ATP levels during aging despite clear changes in mitochondrial morphology (Figure 1E). These results suggest that there are compensatory pathways, like 5' AMP-activated protein kinase, AMPK, that act to maintain a relatively constant ATP level in the muscle, despite alterations in mitochondrial mass and/or structure that could alter mitochondrial function in either beneficial or detrimental ways. Remarkably, despite the actions of these compensatory pathways, the muscles of day 5 and day 7 *daf-2* mutant animals do appear to have elevated ATP levels. This difference occurs at a time in aging when the *daf-2* mutants show their peak mitochondrial mass and along with preserved mitochondrial structure, so it is possible that the muscle mitochondria of the *daf-2* mutant animals generate additional ATP relative to similarly aged wild-type animals. The increases in cellular ATP levels could then provide the additional energy needed to promote enhanced cellular maintenance or other biologic processes that could delay muscle aging. It is also possible that it is increased mitochondrial respiration, in contrast to increased ATP levels, which accounts for the observed benefits. This could involve effects such as mitohormesis, resulting from low levels of reactive oxygen species produced by the mitochondria, or the regeneration of NAD^+^ from NADH. In summary, while the *daf-2* mutants are known to have enhanced mitochondrial function during aging compared to wild-type animals [21], our findings suggest that muscle is at least one of the tissues showing this benefit, and also that the enhanced mitochondrial function can lead to increases in cellular ATP levels.

### The enhanced mitochondrial mass and function of *daf-2* mutants depends on *daf-16*

**Figure 3:**
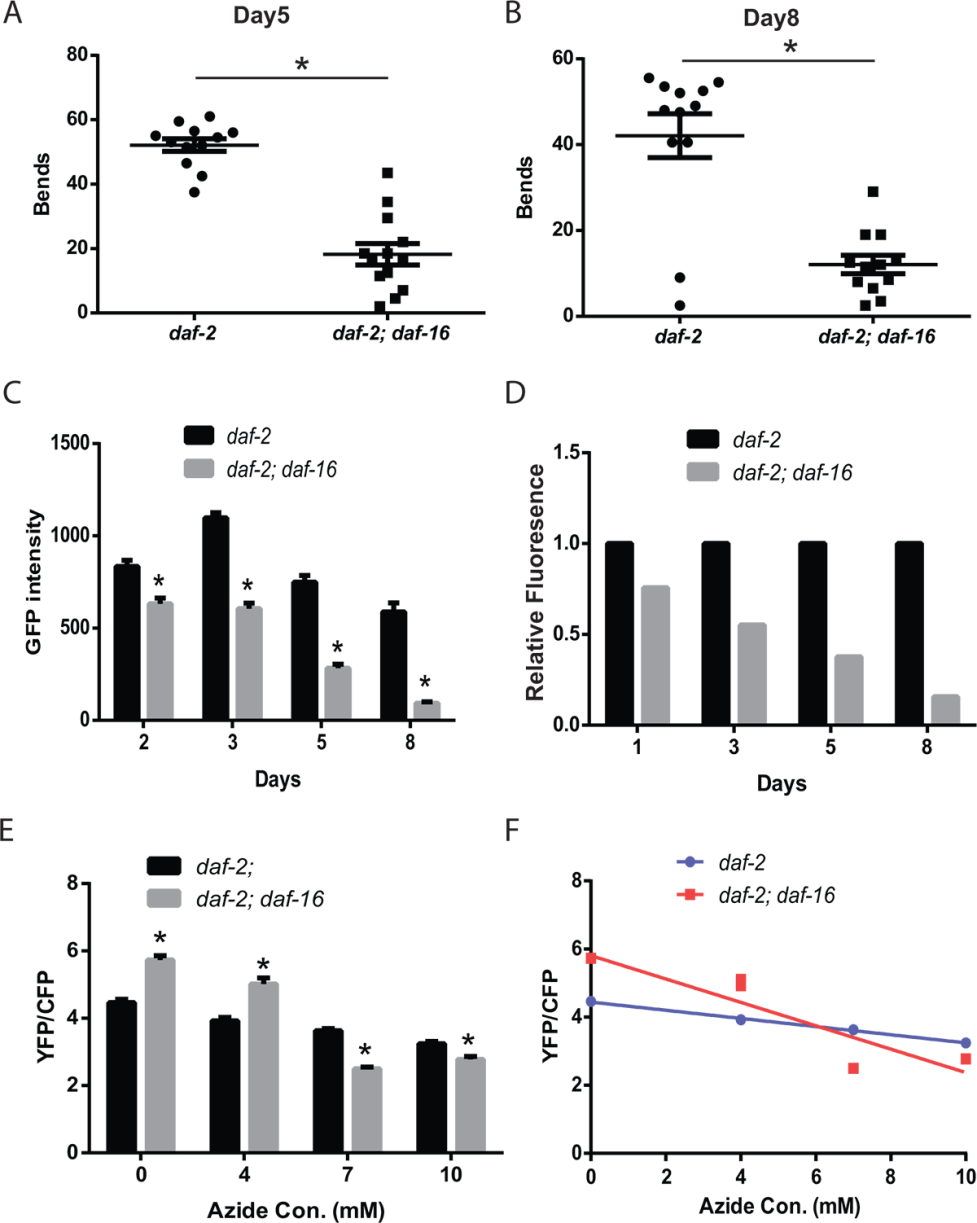
**The *daf-2* mutants exhibit *daf-*16-dependent increases in muscle function and mitochondrial mass.** *daf-2* mutant animals exhibit increased thrashing behavior in liquid, compared to *daf-2 daf-16* mutants, as shown by the measurement of the number bends during a 30 second period on either adult day 5 (**A**) or day 8 (**B**). N = 12 for both genotypes and ages. * represents *p* < 0.05 by *t*‐test. (**C**) The *daf-2 daf-16* mutants show a lower mitochondrial mass and larger age-related decline as shown by the digital imaging and quantitation of animals expressing a mitochondrial-localized GFP. N >12 for all genotypes and ages. * represents *p* < 0.05 by *t*‐test. The declines in mitochondrial mass are particularly evident when the data in (**C**) is replotted to set the *daf-2* fluorescence on each data to 1.0 (**D**). (**E**) The *daf-2 daf-16* mutants show less mitochondrial reserve when activity is reduced by treatment with the mitochondrial inhibitor sodium azide as shown by the imaging of the ATeam ratiometric reporter. Each bar represents the ATeam YFP/CFP ratio from *daf-2* or *daf-2 daf-16* mutant animals treated with the indicated concentration of sodium azide. N = 60 - 70 worms per genotype and treatment. * represents *p* < 0.05 by *t*‐test. (**F**) The differing declines in muscle ATP levels produced by the azide treatment can also be seen when the data from (E) is replotted as an X-Y graph.

The effects of *daf-2* mutations on lifespan require the *daf-16*/FOXO transcription factor which translocates to the nucleus and activates gene expression when *daf-2* activity is low [28–33]. We have previously shown that the preserved mobility of the *daf-2* mutants on solid media during aging also requires *daf-16* [17]. To test whether the preserved mobility in liquid was also *daf-16* dependent, we compared the thrashing movement of aging *daf-2* and *daf-2 daf-16* mutants. We found a clear requirement for *daf-16*, because on both adult day 5 and day 8, the *daf-2 daf-16* mutants showed reduced movement compared to the *daf-2* mutants (Figure 3A and 3B).

We then examined whether *daf-16* contributed to the enhanced muscle mitochondrial mass of the *daf-2* mutant animals. When we measured the expression of a mitochondrial-localized GFP in the muscle of *daf-2* and *daf-2 daf-16* mutants, we found that the *daf-2 daf-16* mutants showed both a lower peak mitochondrial mass and a steeper decline in mitochondrial mass with aging (Figure 3C). The steeper decline was particularly evident when the data from Figure 3C were replotted to directly compare the relative fluorescence of the *daf-2 daf-16* mutants relative to the *daf-2* mutants on each day. This revealed that by adult day 8, the *daf-2 daf-16* mutants retained only a small fraction of the mitochondrial mass of the *daf-2* mutants (Figure 3D). These findings suggest that *daf-16* plays an important role in the effects of *daf-2* mutations on muscle mitochondrial mass both during young adulthood and during aging.

We then asked if the loss of *daf-16* in the *daf-2* mutants led to changes in myocyte ATP levels with the ATeam reporter. We unexpectedly found that adult day 5 *daf-2 daf-16* mutants showed a greater mVenus to mseCFP ratio compared to the *daf-2* mutants which is suggestive of higher ATP levels in the *daf-2 daf-16* mutant compared to the *daf-2* mutant (Figure 3E). The reason for the unexpected increase is unclear but could reflect either an increase in ATP production, particularly pathways acting independent from the mitochondria like glycolysis, or a decrease in ATP consumption, perhaps related to the decreased locomotor activity of the day 5 *daf-2 daf-16* mutants (Figure 3A). Specifically, prior work has shown that periods of *C. elegans* swimming can result in a reduction in whole animal ATP levels [34]. To attempt to uncouple the changes in activity between the mutants, we tested the effects of sodium azide on the ATeam reporter in both strains. We found that adult day 5 *daf-2 daf-16* mutants showed a greater mVenus to mseCFP ratio, consistent with higher ATP levels, when treated with low levels of azide compared to the *daf-2* mutants (Figure 3E and Figure 3F). However, at higher concentrations of azide which resulted in paralysis of the animals, we found that the *daf-2* mutants now showed a greater mVenus to mseCFP ratio, which could suggest that the *daf-2* mutants exhibit higher muscle ATP levels under these conditions. These findings could alternatively be the result of other differences between the *daf-2* and *daf-2 daf-16* mutants such as a greater reserve of mitochondrial capacity due to the greater mitochondrial mass, which might be less sensitive to inhibition by azide, or the activity of one or more of the multitudes of biochemical pathways that influence ATP levels.

### *daf-16* affects muscle mitochondria via both cell autonomous and non-autonomous pathways

**Figure 4:**
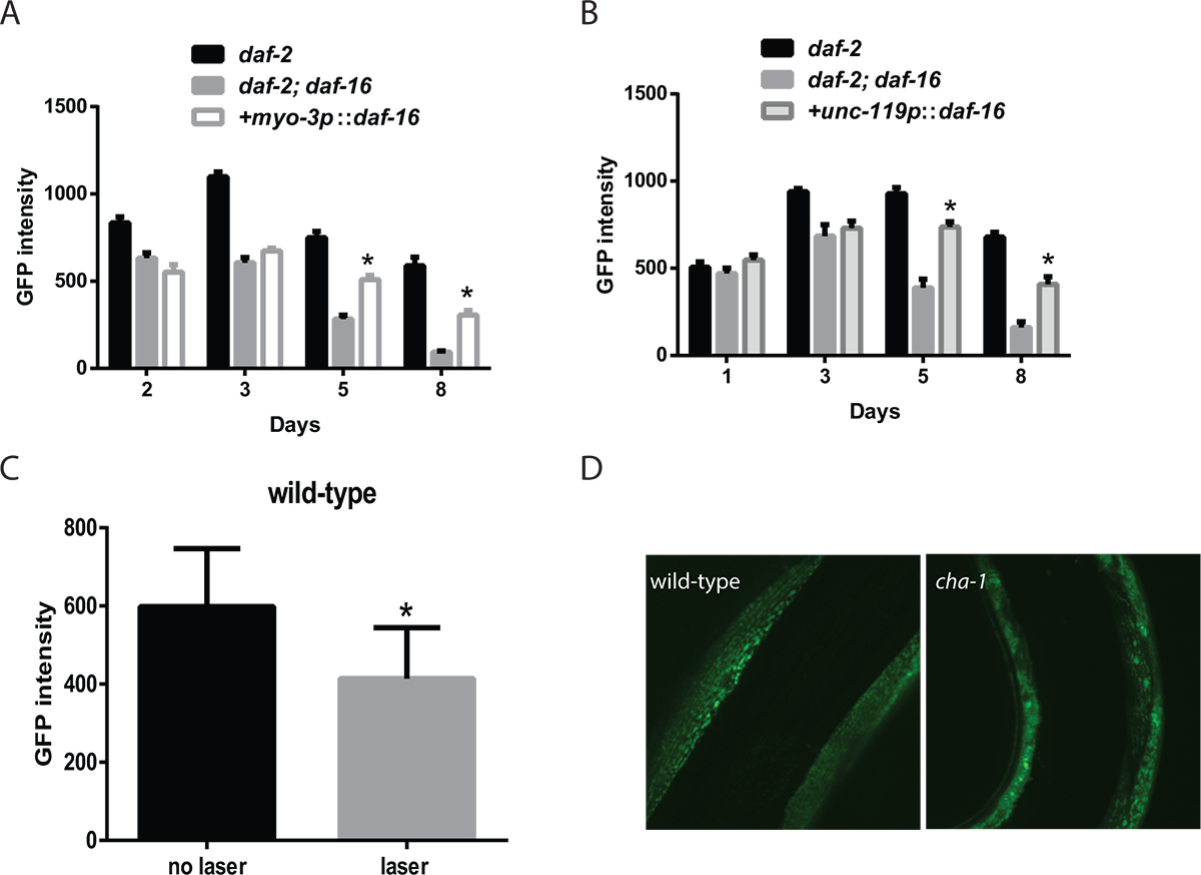
**Innervation and cholinergic signaling alters the muscle mitochondrial mass and structure.** Transgenes expressing *daf-16* in either the muscle (**A**) or nervous system (**B**) were introduced into a *daf-2 daf-16* mutant and the effects on muscle mitochondrial mass were assessed. The restoration of *daf-16* in either the muscle (**A**) or neurons (**B**) can both partially restore the mitochondrial mass of the *daf-2* mutant measured by the fluorescence from a muscle-expressed mitochondrial-localized GFP on the indicated days of adulthood. N >12 for all ages and genotypes. * represents p < 0.05 by *t*‐test. (**C**) The severing of motor neuron commissures via laser axotomy produces a decline in muscle mitochondrial mass in the denervated muscles as shown by the imaging and quantitation of a mitochondrial-localized GFP. * represents p < 0.05 by *t*‐test. (**D**) Disruption of cholinergic signaling in a *cha-1* mutant produces aging-like changes in mitochondrial structure including the formation of abnormal GFP+ aggregates. Shown are confocal images from adult day 3 wild-type and *cha-1* mutants grown at the semi-permissive temperature of 22.5ºC captured from animals expressing a mitochondrial-localized GFP to label the muscle mitochondria.

Via the use of tissue-specific RNAi, we previously demonstrated that the enhanced mobility of *daf-2* mutants required *daf-16* activity in the muscle [17]. However, more recent work has suggested that declines in the function of the motor nervous system precedes declines in muscle function, and these age-related changes in the neurons are also delayed in *daf-2* mutants [16]. These findings suggest that *daf-16* could have effects on muscle function and perhaps even mitochondrial function via effects in the muscle and/or the motor nervous system. To examine the role(s) for *daf-16* activity in both tissues, we used transgenes that restore *daf-16* in neurons or muscle in a *daf-2 daf-16* mutant. Unexpectedly, the rescue of *daf-16* in either the muscle or the nervous system was able to enhance muscle mitochondrial mass in aging *daf-2 daf-16* mutants (Figure 4A and 4B). These effects were particularly notable in both adult day 5 and day 8 animals which are also the time points when the greatest declines in muscle mitochondrial mass in *daf-2 daf-16* mutants relative to the *daf-2* mutants are seen (Figure 4A and Figure 4B). However, the rescue of *daf-16* in either tissue did not fully promote the enhanced muscle mitochondrial mass seen in the younger adult day 3 *daf-2* mutants (Figure 4A and 4B). While this finding could simply reflect that the transgene is only partially active in some way relative to the native *daf-16* gene, it is alternatively possible that the full increase in muscle mitochondrial mass in younger animals could involve *daf-16* activity in other tissues or the combined effects of *daf-16* in both the muscle and nervous system. During aging, we continue to see both cell autonomous actions in the muscle and non-cell autonomous actions in the nervous system that contribute to the increase in muscle mitochondrial mass in the *daf-2* mutants.

We hypothesized that either contact between the motor neuron and the muscle, or even the level of motor neuron activity during aging could contribute to the non-cell autonomous effects of neuronal *daf-16* expression on muscle mitochondrial mass. To determine if contact between the motor neuron and the muscle affected the muscle mitochondrial mass, we performed laser axotomy on several cholinergic commissures in the anterior part of the worm. We visualized these neurons with a transgene expressing GFP under the control of the *acr-2* promoter. Following the cutting of an anterior commissure, we allowed the animals to recover for 24 hours and then measured the expression of the mitochondrial-localized GFP in the now denervated muscle. We found that after axotomy there was a significant reduction in muscle mitochondria mass in the muscle from animals that underwent surgery compared to the sham controls (Figure 4C). This suggested that the innervation of muscles influenced the mitochondrial mass in the innervated muscle.

Among the effects of the axotomy procedure on the innervated muscles would be a reduction in cholinergic signaling from the neuron to the innervated muscle. To assess whether this reduction could affect the muscle mitochondria, we used a *cha-1* mutant to selectively reduce the synthesis of acetylcholine by the motor neurons without affecting other aspects of motor neuron innervation, such as cell-cell contact. The *cha-1* gene encodes the worm ortholog of choline acetyltransferase, and mutations in this gene have been shown to markedly reduce acetylcholine levels in mutant animals [35]. We crossed the temperature-sensitive *cha-1(y226)* mutation into worms carrying the muscle mitochondrial reporter, and then examined the effects of growth at 22.5ºC, which is a non-permissive temperature for the *cha-1* mutation. Adult day 3 animals grown at this temperature showed increased fragmentation and the formation of large abnormal mitochondria aggregates compared to wild-type animals (Figure 4D). These data indicate a link between cholinergic signaling from the motor neuron to the muscle that can alter the structure of the muscle mitochondria.

### *daf-16* acts in the muscle to enhance muscle ATP levels and muscle function

**Figure 5:**
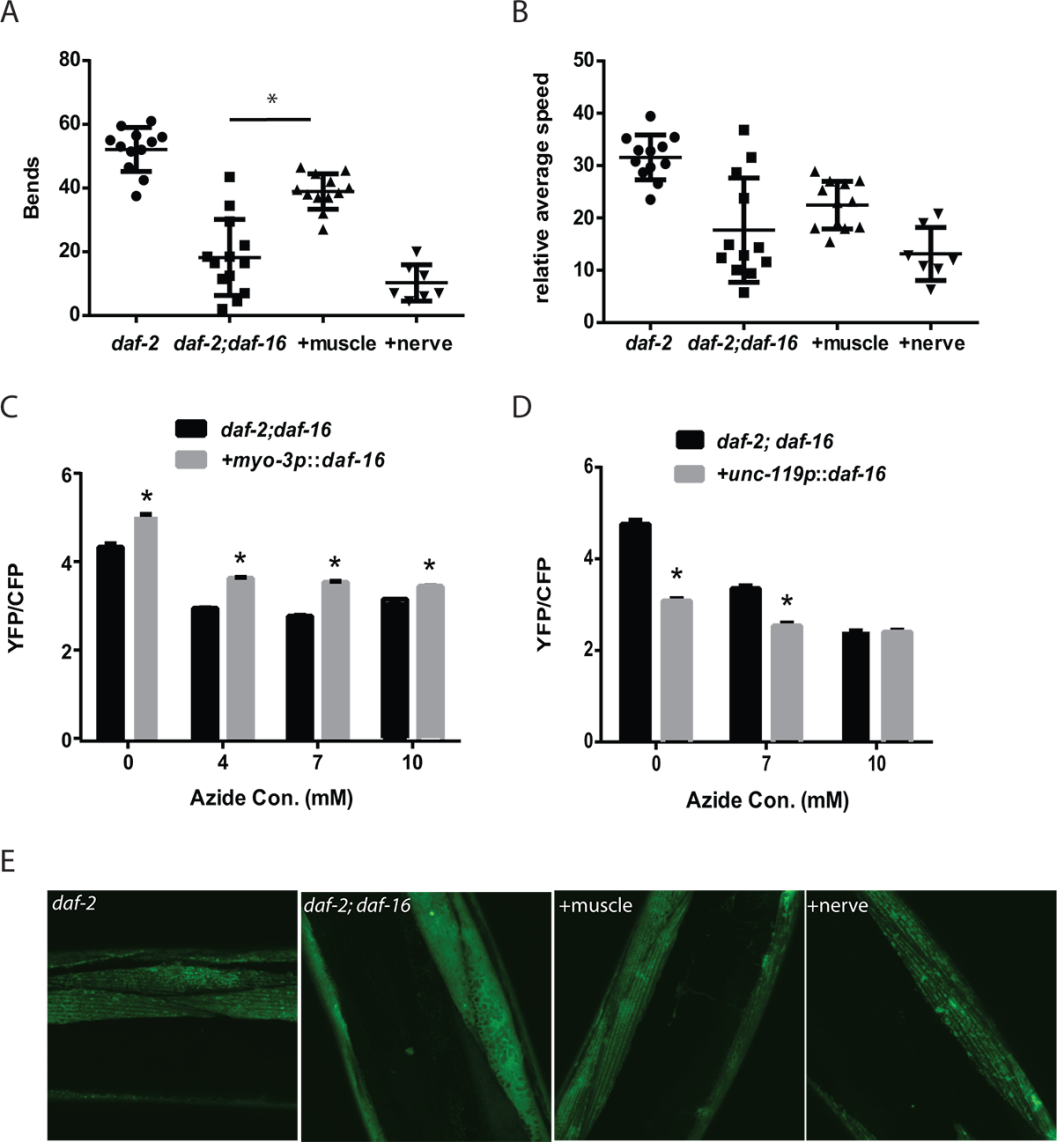
**Cell autonomous and non-cell autonomous actions of *daf-16* in the muscle and nervous system of *daf-2* mutant animals.** Transgenes expressing *daf-16* in either the muscle or nervous system were introduced into a *daf-2 daf-16* mutant and the effects on mobility as shown by thrashing behavior in liquid was assessed. Shown are bar graphs depicting either the average number of body bends (**A**) or relative speed (**B**) over a 30 second period by adult day 5 *daf-2*, *daf-2 daf-16,* or *daf-2 daf-16* mutants with *daf-16* restored in the muscle (+muscle) or neurons (+nerve). N = 12 for all genotypes. * represents *p* < 0.05 by *t*‐test. Moreover, the rescue of *daf-16* in the muscle (**C**), but not the neurons (**D**), is able to restore the increased mitochondrial activity of the *daf-2 daf-16* mutants as shown by treatment with increasing doses of the mitochondrial inhibitor sodium azide. N = 60 - 70 worms per genotype and treatment. * represents *p* < 0.05 by *t*‐test. (**E**) While the rescue of *daf-16* in both the muscle and neurons increased mitochondrial mass, only rescue in the muscle (+muscle) improved mitochondrial structure in day 5 animals compared to *daf-2 daf-16* mutants or animals with *daf-16* restored in the nervous system (+nerve) as shown by confocal images of muscle mitochondria.

While *daf-16* can enhance muscle mitochondrial mass via actions in either the muscle or nervous system, it is unclear whether these effects translate into either enhanced mitochondrial function or improved muscle function during aging. To test whether the rescue of *daf-16* in either the muscle or the nervous system was able to increase muscle function in aging *daf-2 daf-16* mutants, we compared the thrashing movement of aging *daf-2* and *daf-2 daf-16* mutants to *daf-2 daf-16* mutants where *daf-16* has been restored in either tissue with a transgene. In contrast to the effects on mitochondrial mass, we found that only the restoration of *daf-16* in muscle was able to partially rescue the preserved swimming behavior of adult day 5 *daf-2* mutants (Figure 5A and Figure 5B).

We also examined the effects of tissue-specific *daf-16* rescue on muscle ATP levels using the ATeam reporter. We found that the restoration of *daf-16* in the muscle did enhance muscle the mVenus to mseCFP ratio which suggests an increase in muscle ATP levels (Figure 5C), whereas the YFP/CFP ratio was lower when *daf-16* was restored in the nervous system. However, due to concerns regarding the differences in mobility between the *daf-2 daf-16* mutants and particularly the animals where *daf-16* is restored in the muscle, we again used the mitochondrial inhibitor sodium azide as an attempt to reduce the potential effects of physical activity on ATP levels. In the animals expressing *daf-16* in the muscle, we found that azide reduced the YFP/CFP ratio consistent with a decline in ATP levels, but the YFP/CFP ratio was always greater than in the *daf-2 daf-16* control animals (Figure 5C). In contrast, a similar effect was not seen in animals expressing *daf-16* in the nervous system (Figure 5D). Our results suggest that despite the increase in mitochondrial mass seen after *daf-16* was restored in either tissue (Figure 4A and 4B), only the expression of *daf-16* in the muscle was able to lead to enhanced muscle mitochondrial function.

Consistent with this possibility, confocal images of muscle mitochondria in transgenic animals expressing a mitochondrial-targeted GFP reveal that on adult day 5, only the mitochondria of the animals expressing *daf-16* in the muscle exhibit a preservation of mitochondrial morphology compared to either *daf-2 daf-16* mutant used as a reference or animals where *daf-16* is restored in the nervous system (Figure 5E). In particular, the muscle mitochondria from the transgenic animals expressing *daf-16* in the muscle show preservation of the alignment and tubular structure akin to those seen in the *daf-2* mutant. In contrast, the expression of *daf-16* in the nervous system is not sufficient to delay the onset of mitochondrial fragmentation (Figure 5E).

### Reduced *daf-2*/IGFR signaling can compensate for reduced neuronal activity

**Figure 6:**
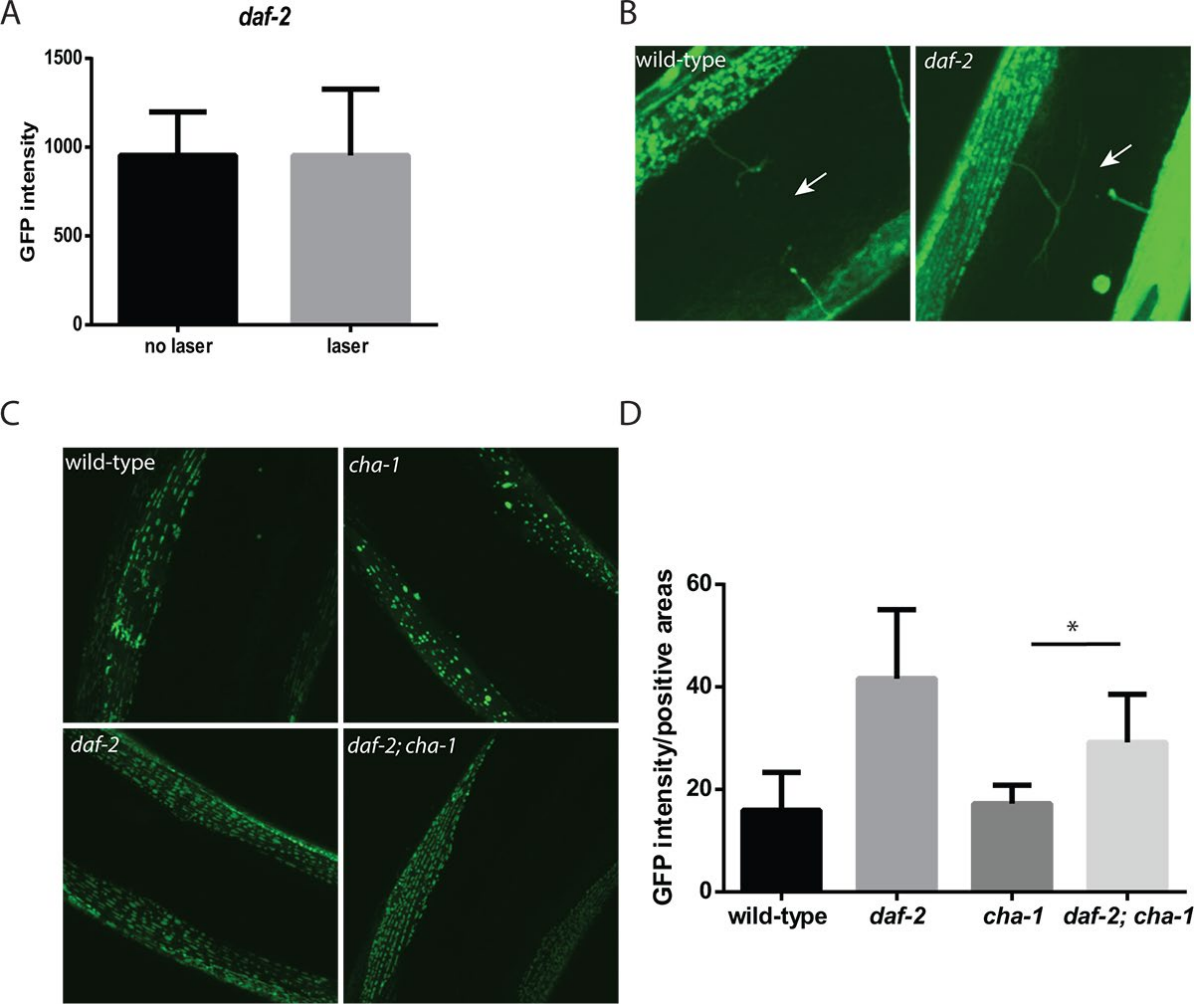
***daf-2* mutations can protect *C. elegans* muscle from the effects of denervation or reduced cholinergic signaling.** (**A**) In contrast to experiments performed in wild-type animals, the severing of motor neuron commissures via laser axotomy in *daf-2* mutant animals did not reduce muscle mitochondrial mass in the denervated muscles as measured by fluorescence from a mitochondrial localized GFP. N >12 for both genotypes and treatments. (**B**) The differing effects were not simply due to the regrowth of the axons in the *daf-2* mutants as both the wild-type (left image) and *daf-2* mutants (right image) showed the presence of severed axons as indicated by the arrows. Both images were taken with confocal microscopy using a 100X oil-immersion objective. (**C**) The *daf-2* mutation is able to rescue the effects of the *cha-1* mutation on muscle mitochondrial structure. Shown are confocal images from adult day 15 wild-type, *cha-1*, *daf-2*, and *daf-2 cha-1* mutants, expressing a mitochondrial-localized GFP to label the muscle mitochondria, grown at the permissive temperature 16ºC. These images show the age-related disruption of the filamentous mitochondrial structure in the wild-type animals which is reduced in the *daf-2* mutants. Additionally, the chronic low-level disruption of cholinergic signaling in the *cha-1* mutant even at permissive temperature exacerbates the disruption of muscle mitochondrial structure. However, the *daf-2* mutation is also able to attenuate this decline in mitochondrial structure. (**D**) These declines can also be visualized by measuring the GFP+ area in the myocyte containing the mitochondria relative to the total muscle area in the confocal images. N >5 for all genotypes. * represents *p* < 0.05 by *t*‐test.

Earlier we demonstrated that the laser axotomy procedure performed on wild-type animals led to a significant reduction in the muscle mitochondria mass in the formerly innervated muscles (Figure 4C). We then tested the effects of the same procedure when performed on *daf-2* mutant animals expressing a mitochondrial localized GFP in the muscle. In contrast to wild-type animals, performing the axotomy procedure on the *daf-2* mutants resulted in little change in the mitochondrial mass (Figure 6A). Since prior work has shown that *daf-2* mutant animals demonstrate enhanced axonal regeneration compared to wild-type animals, we assessed the degree to which the cholinergic neurons had regenerated in the *daf-2* mutants to ensure that our results did not simply reflect reinnervation of the muscles [36]. When we examined the motor neurons of animals at the 24 hour time point, we found that both the wild-type animals and *daf-2* mutants showed evidence of axonal regeneration, but an intact axon did not yet appear to be restored (Figure 6B). This suggested that the reduced *daf-2* signaling in the *daf-2* mutants could somehow protect the muscle mitochondria from the effects of denervation.

To understand whether the protective effects of reduced *daf-2* signaling also extended to reductions in cholinergic signaling, we again used the *cha-1* mutant to reduce acetylcholine levels while retaining other aspects of innervation such as cell-cell contact. We grew the *cha-1* mutant at the semi-permissive temperature of 16ºC. At this temperature, the animals show grossly normal movement suggesting that the acetylcholine levels are reduced but not truly absent like when the animals are shifted to non-permissive temperatures, like 25ºC. We found that at this temperature, although the adult day 15 wild-type animals showed declines in mitochondrial structure due to aging, similarly aged *cha-1* mutants showed further declines in mitochondrial structure and also exhibited the formation of large abnormal mitochondria (Figure 6C). In contrast, both the day 15 *daf-2* mutants and the day 15 *daf-2 cha-1* mutants showed enhanced mitochondrial structure with the *daf-2 cha-1* mutants showing a marked reduction in the formation of abnormal mitochondria compared to the *cha-1* mutants alone (Figure 6C). We attempted to quantify the declines in the mitochondrial network by measuring the fraction of the muscle area that was occupied by GFP expressing mitochondria (Figure 6D). These measurements demonstrated that the *daf-2* mutation can increase the mitochondria density of both wild-type and the *cha-1* mutant animals. Together these results suggest that reductions in neuronal acetylcholine release can directly alter the number and structure of the muscle mitochondria, and that in the *daf-2* mutants the reduction in *daf-2* signaling, and perhaps activation of *daf-16*, can shield the muscle mitochondria from these adverse effects.

## DISCUSSION

### Muscle function and mitochondrial function are linked during *C. elegans* aging

Our study explored the effects of aging and changes in *daf-2*/IGFR activity on muscle function and mitochondrial activity during aging. While studies using samples from either humans or aging rodents have suggested several adverse effects of aging on mitochondrial structure and function, our use of *C. elegans* allowed us to visualize and track the numbers, structure, and function of the muscle mitochondria in vivo. Additionally, via the use of interventions, such as a hypomorphic *daf-2* mutant that both extends worm lifespan and leads to preserved mobility during the early stages of the aging process [14,15,17], we have been able to examine whether changes observed during aging respond to alterations in *daf-2* activity or other interventions. The ability to use these interventions helps to discern the linkage between an individual age-related change and the possible downstream effects on muscle function with aging.

Via this approach, we found that age-related losses in muscle function can be partially rescued by compounds that can enhance the activity of dysfunctional mitochondria (Figure 1A) which suggests that muscle aging in *C. elegans* could be the result of a decline in mitochondrial structure and function. Consistently, the *daf-2* mutant animals show an increase in mitochondrial mass in both young animals and also aging worms (Figure 1C). In addition, we found that the structure of the mitochondria remains preserved during aging in the *daf-2* mutant animals (Figure 1E). The muscle mitochondria of young adult worms form a tubular network that runs parallel to the myosin fibrils in the muscle. During aging, this tubular structure declines, and is accompanied by an increase in mitochondrial fission. Reduced *daf-2* signaling preserves this structure in a *daf-16*-dependent manner (Figure 5E). Additionally, the *daf-2* mutants exhibit increased cellular ATP levels as shown via the use of the ATP-sensitive ratiometric reporter ATeam (Figure 2). However, it is not clear how DAF-16 acts to preserve mitochondrial structure in an aging context. In young worms, DAF-16 acts to promote the tubular structure of mitochondria via EAT-3 mediated mitochondrial fusion [34]. During aging in *daf-2* mutants, DAF-16 may continue to act via EAT-3, or could instead couple to alternate mechanisms, such as increased mitochondrial biogenesis or enhanced autophagy that could reduce the pool of damaged mitochondria.

While our results do not show a clear cause and effect between enhanced mitochondrial function and alterations in mobility and muscle function, the linkage between preserved mitochondrial structure and preserved mobility, and the ability to enhance mobility by drug treatments intended to enhance mitochondrial activity, does suggest an important role for mitochondria in *C. elegans* muscle aging. The beneficial effects of *daf-16* on the muscle mitochondria also must extend beyond simply boosting the mitochondrial mass. The restoration of *daf-16* in either muscle or the nervous system can increase the muscle mass during aging (Figure 4A and 4B), but only the restoration in the muscle is able to enhance the reserve of muscle mitochondrial activity (Figure 5C). This later effect is linked to enhanced muscle function during aging which is only seen when *daf-16* activity is restored in the muscle. Perhaps this suggests that *daf-16* acts specifically in the muscle to both increase mitochondrial mass and to enhance the activity of some or all mitochondria via currently unknown additional mechanism(s).

### DAF-16/FOXO enhances mitochondrial function via cell autonomous and non-cell autonomous pathways

An unexpected finding of our work is that the restoration of *daf-16* in either the nervous system or the muscle is able to enhance the muscle mitochondrial mass of *daf-2 daf-16* mutants (Figure 4A and 4B). However, only the restoration of *daf-16* in the muscle is able to restore the preserved mobility during aging to *daf-2 daf-16* mutants. This latter finding is consistent with our prior work showing that the inhibition of *daf-16* using muscle-specific RNAi reduces the mobility of aging *daf-2* mutants [17]. However, this finding also extends our work by showing that *daf-16* activity in the muscle is sufficient to enhance mobility in a *daf-2* mutant.

Prior work has demonstrated that *daf-16* acts in multiple tissues downstream of *daf-2* to promote the effects of *daf-2* on lifespan and dauer formation. Experiments using tissue-specific transgenes to restore *daf-16* in a *daf-2 daf-16* mutant has demonstrated that *daf-16* primarily acts in the worm intestine to promote the enhanced longevity of *daf-2* mutants [37]. In contrast, the restoration of *daf-16* in the nervous system only produces a modest increase in longevity and the restoration in the muscle produces almost no effect on lifespan [37]. However, for the promotion of dauer development, *daf-16* acts in the nervous system and not in the other tissues. The mechanism(s) by which *daf-16* activity in a single tissue then produces animal-wide phenotypes, like enhanced longevity or dauer development, is still not fully understood.

Our data suggests an interaction between *daf-16* activity in the nervous system and the muscle that leads to the enhanced mitochondrial mass of the *daf-2* mutants. However, we also find that the restoration of *daf-16* only in the nervous system is able to enhance the mitochondrial mass but not necessarily the mitochondrial function of the *daf-2* mutants (Figure 4B and Figure 5D). We subsequently show that cholinergic signaling between the motor neuron and the muscle promotes the mitochondrial mass or mitochondrial structure in the muscle as both denervation and blocking acetylcholine synthesis with *cha-1* mutants can disrupt these in the muscle. While denervation reduces mitochondrial mass as measured by fluorescence (Figure 4C), we do not see that effect in the *cha-1* mutants. However, we suspect that this could reflect the masking of any declines by the strong GFP signal produced by the large, abnormal mitochondria generated in the *cha-1* mutants (Figure 4D). During aging, it is possible that the restoration of *daf-16* in the nervous system enhances neuronal structure or activity in some way to promote cholinergic signaling to the muscle. Consistently, *daf-2* mutants have been shown to have increased neuronal activity during aging [16].

However, *daf-2* mutations can also act to protect muscle mitochondria either from the adverse effects of denervation or reduced cholinergic signaling (Figure 6A, Figure 6C, and Figure 6D). Additional work will be needed to determine if this effect requires the *daf-16*/FOXO transcription factor and whether the effect occurs directly in the muscle or is mediated indirectly through another tissue.

An important cell autonomous effect of *daf-16* in the setting of muscle aging is the preservation of muscle mitochondria structure and muscle activity during aging in *daf-2* mutants along with the preservation of muscle function (Figure 5A and Figure 5C). It is currently unclear if these two effects are linked with the improvements in mitochondrial function in some way spurring beneficial effects in regards to muscle structure, contractile activity, or another parameter. Our finding that drugs shown to enhance the activity of dysfunctional mitochondria can enhance the mobility of aging animals does provide support for this mechanism (Figure 1A). However, it is also possible that *daf-16* acts via a distinct mechanism that has the combined net effect of preserving mitochondrial and muscle function during aging.

## MATERIALS AND METHODS

### *C. elegans* strains and maintenance

The following *C. elegans* strains were used in this study were obtained from the *C. elegans* Genetics Center which is funded by NIH Office of Research Infrastructure Programs (P40 OD010440): N2, CZ631 (juIs14[acr-2p::GFP + lin-15(+)]), DR1568 (*daf-2 (e1371)*), GR1307 (*daf-16(mgDf50)*), HT1593 (*unc-119(ed3)*), SJ4103 (*zcIs14 [myo-3::GFP(mit)]*), TJ1060 (*spe-9(hc88); fer-15(b26)*), and TY1652 (*cha-1(y226)*). The strains NQ145 (*daf-16(mgDf50); qnEx38[Pmyo-3:GFP::daf-16; Pmyo-2:mCherry]*) and NQ440 (*daf-16(mgDf50); qnIs42[Punc-119:GFP::daf-16; Pmyo-2:mCherry]*) were kindly provided by Dr. David M. Raizen [38]. GL227 (*daf-2(e1371); spe-9(hc88); fer-15(b26)*) was described previously [17]. The *qnEx38* transgene was integrated via UV-irradiation and then outcrossed with N2 to give rise to *bafIs21[Pmyo-3:GFP::daf-16; Pmyo-2:mCherry]*. Double mutants were generated by standard genetic crosses, and the genotypes of strains were confirmed by PCR using oligos which detect gene deletions or point mutations.

All *C. elegans* strains were propagated on standard nematode growth agar (NGA) plates containing streptomycin (200 µg/mL) and spotted with OP50-1, as previously described [39]. For RNAi experiments, worms were fed OP50(xu363), which is an OP50-derived bacterial strain designed to deliver RNAi [40].

### Generation of transgenic animals

ALF22 (*bafIs22[myo-3p::GFP::myo-3; unc-119^+^]*) was constructed *via* the microparticle bombardment of HT1593 with previously described fosmid modified by recombineering to insert the sequence for GFP at the N-terminus of the MYO-3 protein coding sequence [41,42]. This transgene expresses a GFP::MYO-3 fusion protein which is similar in sequence and arrangement to that expressed by the RW1596 (*myo-3(st386) V; stEx30*) strain, which has been shown to integrate into muscle myofibrils and successfully rescue the *myo-3* mutation [43]. Prior to use the *bafIs22* transgene was backcrossed three times with N2.

ALF23 (*bafIs23[myo-3p::ATeam; unc-119^+^]*) was generated by the microparticle bombardment of HT1593 with a plasmid carrying a *myo-3p::ATeam* transgene and also retrofitted by homologous recombination to carry the *unc-119* gene from *C. briggsiae* as a selectable marker. The cDNA for the FRET-based ATP ratiometric reporter [18] with *C. elegans* optimized codon usage was synthesized, and then cloned into pPD93.97 (Addgene plasmid #1476) as a SacI-EcoRI fragment. The resulting plasmid was then modified by homologous recombination to carry the *unc-119* marker as previously described [44]. Transgenic animals were identified *via unc-119* rescue as well as visible YFP fluorescence, and then backcrossed three times with N2 prior to use.

### Motility assay

Animals were synchronized by hypochlorite treatment and then grown at 20°C until L4 and then maintained at 25°C for the remainder of the experiment. Depending upon the experiment, worms were either grown on OP50-1 bacteria or OP50(xu363) to deliver either control or the indicated RNAi. For mitochondrial activator treatment, worms were grown from egg hatching on OP50-1 until day 1 of adulthood when they were transferred to OP50-1 spotted plates containing 2.6mM riboflavin or 75mM methylene blue. The doses of both drugs were determined from pilot experiments using escalating doses from prior work using either riboflavin or methylene blue in *C. elegans*. In the prior work each drug was shown to selectively benefit mutant animals expressing either a mutant NUO-1 subunit of complex I or mutant TDP-43 and FUS proteins associated with neurologic diseases, respectively [19,45]. The beneficial doses reported in these papers had little effect on wild-type animals which could suggest that the mutants are selectively sensitive to lower doses than aging wild-type animals.

On days 1, 3, 5, and 7 of adulthood, random worms were selected and placed individually in a well of a 12-well tissue culture plate filled with room-temperature S-basal. The worms were allowed to swim undisturbed for 30 seconds to acclimate after being transferred and to dislodge any adhering bacteria. At this point, worm movement was digitally recorded for 30 seconds using an ImagingSource digital camera mounted on a Zeiss Stemi 2000-C microscope and the ImagingSource IC Capture 2.3 software. Videos were recorded at 15 frames per second, and the captured frames were merged into the *.avi* format and imported directly into ImageJ. The thrashing rate was then measured using the wrMTrck plugin in ImageJ (available at www.phage.dk/plugins/wrmtrck.html) [46]. One body bend was defined as complete bending of the worm body in one direction to the outermost angle and back to the initial posture. Thrashes were counted from 10 to 12 worms per treatment condition.

### Microscopy

Worms carrying the *zcIs14[myo-3::GFP(mit)]* transgene were mounted on the indicated days on a 2% agarose pad and immobilized using 10 mM levamisole hydrochloride to avoid disruption of mitochondrial function. Mounted worms were digitally imaged using a Nikon Eclipse Ti inverted microscope and CoolSNAP ES2 digital camera using a Nikon Endow GFP filter set as previously described [47,48]. Up to 15 animals were imaged per group. Muscle GFP fluorescence intensity was quantified using ImageJ [46]. The fluorescence levels in each group were averaged, and the significance of differences were assessed using Student's *t*-test with *p* < 0.05 being considered a significant difference.

To analyze mitochondrial morphology, worms carrying the *zcIs14[myo-3::GFP(mit)]* transgene were mounted on the indicated days as described above. The muscle mitochondria were then visualized using confocal laser-scanning microscopy. During the confocal microscopy experiments, the exposure settings were adjusted between days and genetic backgrounds to ensure that the details of the mitochondrial morphology were visible despite the overall differences in GFP fluorescence present. These images were not used to assess quantitative changes in fluorescence levels due to aging or genetic mutations.

### Ratiometric imaging

Worms from a synchronized population were mounted on the indicated days on a 2% agarose pad and immobilized using 10 mM levamisole hydrochloride with up to 15 animals per group being imaged. The mounted worms were then imaged using a Nikon Eclipse Ti inverted microscope and CoolSNAP ES2 digital camera. ATeam fluorescence detection was carried out in rapid succession using two separate filter cubes to capture the mseCFP fluorescence directly emitted (435 nm excitation / 475 nm emission) versus coupled to mVenus *via* FRET (435 nm excitation / 527 emission). During imaging, detectors built into the Nikon Elements software were used to establish the exposure settings with the goal of avoiding image saturation. The fluorescence intensity was measured in several regions of interest in each pair of images using ImageJ [46]. The ratio of the fluorescence in the 527 nm emission image divided by the fluorescence in the 427 nm emission image was then calculated and used to represent the relative ATP concentration in the myocytes.

### Laser axotomy and imaging

Adult day 3 and day 4 hermaphrodites were mounted in S-basal containing 10 mM levamisole on a 2% agarose pad under a cover slip. GFP-expressing cholinergic neurons and commissures were monitored with a fluorescence microscope using a Nikon 100X, 1.4 NA lens. Selected commissural axons were cut using a MicroPoint Laser System (Andor Technology). After surgery, animals were placed on a fresh OP50-1 spotted NGA plate, and then remounted for quantitative light microscopy imaging approximately 24-28 hours post-surgery. A minimum of 10 individuals (with 1 axotomized commissure per worm) were observed for most experiments and repeated 3 times.

### Ratiometric spectrofluorometry

Synchronized day 5 adult animals expressing the ATeam ratiometric reporter were washed from plates and transferred in S-basal to wells of a 96-well flat-bottom black plate with each well containing roughly 50-70 worms. S-basal containing a final concentration of 0mM, 4mM, 7mM, or 10mM sodium azide was added immediately prior to loading the plate into a SPECTRAmax Gemini EM spectrofluorometer (Molecular Devices, Sunnavale, CA). After a 5 minute incubation at 25°C, fluorescence was measured over a 30 minute period. Ateam was excited at 435 nm and emission was measured at 475 and 512 nm. Data collected by the fluorometer was then exported to Microsoft Excel for analysis.
